# Impact of the Cachexia Index on survival and recurrence in patients undergoing surgery for gastric cancer

**DOI:** 10.1007/s00595-026-03393-y

**Published:** 2026-07-13

**Authors:** Haruki Kojima, Kotaro Sugawara, Koichi Yagi, Shoh Yajima, Yoshiyuki Miwa, Shuichiro Oya, Asami Okamoto, Raito Asaoka, Tomoki Kaname, Yoshifumi Baba

**Affiliations:** Department of Gastrointestinal Surgery, Graduate School of Medicine, The University of Tokyo, 7-3-1 Hongo, Bunkyo-ku, 113-8655 Tokyo, Japan

**Keywords:** Cachexia index, Gastric cancer, Sarcopenia

## Abstract

**Purpose:**

This study aimed to elucidate the clinical relevance of the cachexia index (CXI), an integrative metric encompassing sarcopenia, nutritional status, and systemic inflammatory burden, in patients undergoing surgery for gastric cancer (GC).

**Methods:**

We retrospectively reviewed 1,166 patients who underwent curative gastrectomy for GC. The CXI was calculated as follows: skeletal muscle index (SMI) × serum albumin concentration (g/dL) / neutrophil-to-lymphocyte ratio (NLR). Patients were dichotomized according to sex-specific lowest quartile cutoff values (61.6 for men and 49.2 for women). The impact of the CXI on overall survival (OS), disease-free survival (DFS), and the recurrence patterns was studied. Multivariate Cox regression analyses were performed to identify any independent predictive factors for a poor survival.

**Results:**

A low CXI was significantly associated with advanced age (≥ 65 years), a higher comorbidity burden, an advanced pathological stage, and a higher incidence of postoperative complications. Patients in the low-CXI group had a significantly poorer OS than those in the high-CXI group (5-year OS: 63.4% vs. 78.5%, *P* < 0.001), with a consistently inferior survival across pathological stages I-III. A low CXI was also significantly associated with a poorer DFS (5-year DFS: 61.0% vs. 76.7%, *P* < 0.001). Among the patients with stage II/III disease, hematogenous recurrence occurred more frequently in the low-CXI group than in the high-CXI group (13.6% vs. 6.9%, *P* = 0.026). A multivariate analysis revealed that a low CXI was an independent predictor of a poor OS (HR 1.58, 95% CI: 1.20–2.07, *P* = 0.001).

**Conclusions:**

The CXI is useful for predicting the survival outcomes of GC patients, irrespective of tumor stage. A low CXI was associated with a higher incidence of recurrence, especially hematogenous recurrence.

**Supplementary Information:**

The online version contains supplementary material available at https://doi.org/10.1007/s00595-026-03393-y.

## Introduction

Gastric cancer (GC) remains a major contributor to cancer-related mortality worldwide and it continues to be highly prevalent in East Asian countries, including Japan [1]. Although the pathological stage is the major prognostic determinant for patients with GC [2], the physiological status of the patient substantially influences postoperative survival following gastrectomy [3–7]. In this context, multiple indices reflecting the nutritional and immunological status, such as the prognostic nutritional index (PNI) [8, 9], nutritional risk index [10], and controlling nutritional status score [7], have been proposed as clinically relevant tools for predicting the outcomes in GC. Beyond isolated nutritional parameters, cancer cachexia has emerged as a complex systemic condition characterized by progressive skeletal muscle loss and chronic inflammation, both of which have been implicated in adverse oncological outcomes in patients with gastrointestinal malignancies [11–13].

The Cachexia Index (CXI) was recently introduced as a composite metric that integrates skeletal muscle mass, nutritional reserve, and inflammatory status into a single quantitative measure [14]. This index captures the multidimensional physiological derangements associated with cancer cachexia. Previous studies have reported that a reduced CXI is associated with an inferior survival in patients with lung cancer, cholangiocarcinoma, and hepatocellular carcinoma [15, 16].

In GC, several investigations have suggested the potential prognostic relevance of the CXI [17–19]. However, the available evidence is limited by modest cohort sizes and insufficiently granular survival analyses, particularly with respect to disease stage, cause-specific mortality, and recurrence patterns. Consequently, the prognostic and clinical implications of the CXI in surgically treated GC remain to be fully elucidated.

We herein investigated the significance of the CXI in a large cohort of patients who underwent surgery for GC. Furthermore, we aimed to reveal the impact of the CXI on the disease-free survival and recurrence patterns.

## Patients and Methods

### Patients

In total, 1697 patients underwent gastrectomy for primary GC at the University of Tokyo Hospital between April 2001 and December 2014. We excluded 162 patients who underwent non-curative surgery, 251 who received endoscopic therapy or preoperative chemotherapy, 58 with concurrent malignancies, 33 who lacked postoperative follow-up within six months, and 27 with incomplete clinical datasets. A total of 1166 patients with prospectively maintained clinical records were included in this study. Follow-up was conducted until June 2025, and the median follow-up duration among survivors was 83.9 months. This retrospective study was approved by the Ethics Committee of the Faculty of Medicine, University of Tokyo (ID: 3962).

## Cachexia Index

The CXI was calculated using the following formula: SMI (cm²/m²) × serum albumin concentration (g/dL) ÷ NLR [14]. The cutoff values for the CXI were determined as the sex-specific lower quartile within this cohort: 61.6 (cm²·g/m²·dL) for men and 49.2 (cm²·g/m²·dL) for women, using a simple and readily applicable criterion that could also be adopted in other studies. SMI measurements were derived from preoperative computed tomography imaging, as described previously [20]. The skeletal muscle area at the L3 level was quantified using the OsiriX software program and normalized to height squared to obtain the SMI.

## Studied criteria

Comorbidity severity was evaluated using the Charlson Comorbidity Index (CCI) [21]. The histological classification followed the Lauren system [22]. Pathological staging adhered to the 7th edition of the TNM classification [23]. Surgical procedures, including the type of gastrectomy and the extent of lymphadenectomy, were based primarily on the Japanese Gastric Cancer Treatment Guidelines [24], with modifications according to individual patient conditions. Postoperative complications were graded using the Clavien-Dindo (C-D) classification system [25]. Recurrence was classified as locoregional, lymph node, peritoneal, or hematogenous [26, 27].

### Statistical analysis

Associations between the CXI and clinicopathological variables were assessed using the Wilcoxon signed-rank test for continuous variables and the chi-square test for categorical variables. Overall survival (OS) was evaluated using the Kaplan–Meier analysis, and intergroup comparisons were performed using the log-rank test. Variables with *P* < 0.05 in the univariate Cox proportional hazards models were included in the multivariate analyses. All statistical analyses were conducted using the JMP Student Edition 18 software program.

## Results

### Patient Characteristics

The high- and low-CXI groups included 874 (612 men and 262 women) and 292 (204 men and 88 women) patients, respectively. The median age was significantly higher in the low-CXI group than in the high-CXI group (70 vs. 65 years, *P* < 0.001). A higher proportion of patients with a CCI ≥ 2 was observed in the low-CXI group (20.9% vs. 11.6%, *P* = 0.001). Patients with a low CXI developed postoperative complications (≥ C-D grade II) more often than those with a high CXI (25.7% vs. 20.5%, *P* = 0.04). The low-CXI group included a higher proportion of patients with advanced-stage GC (≥ pStage III) than the high-CXI group (27.7% vs. 13.2%, *P* < 0.001). Overall, a low CXI was significantly associated with advanced age, severity of comorbidity, occurrence of postoperative complications, and advanced pStage (Table 1).

## The survival difference between the low- and high-CXI groups

**Fig. 1:**
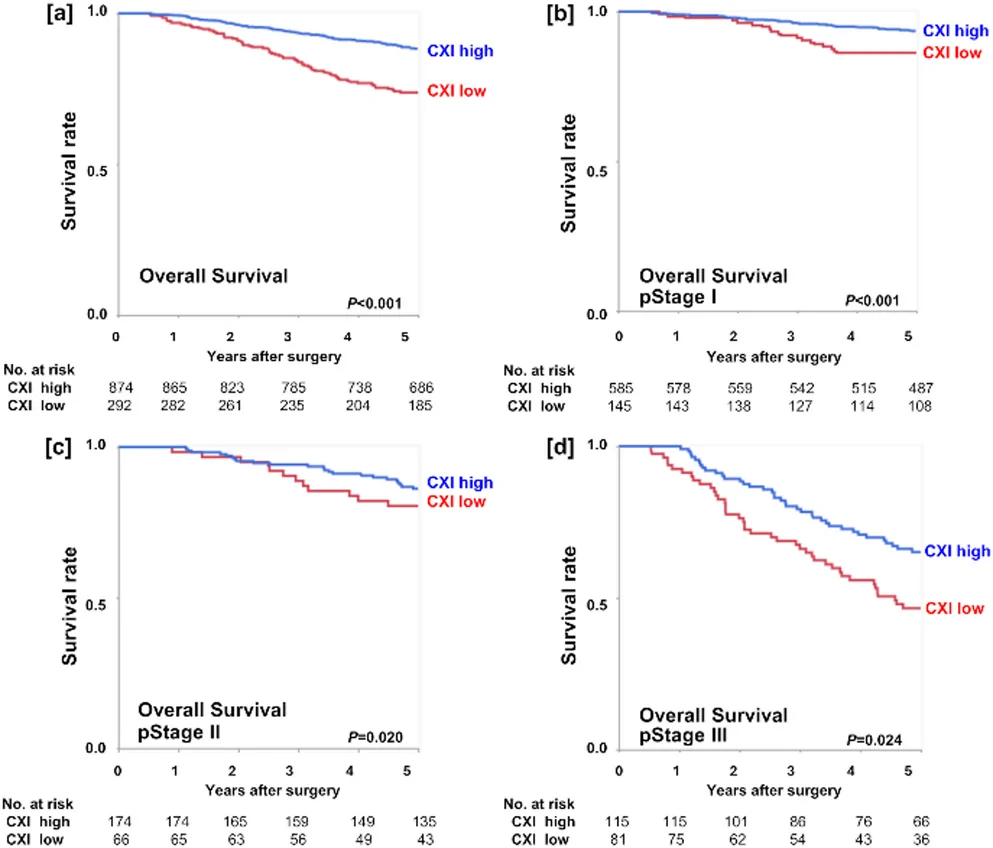
OS outcomes according to the CXI** (a)** Patients in the low-CXI group exhibited a significantly poorer OS than those in the high-CXI group (*P* < 0.001). The survival difference according to the CXI was also found in patients with **(b)** pStage I GC (*P* < 0.001), **(c)** pStage II GC (*P* = 0.020), and **(d)** pStage III GC (*P* = 0.024)

Patients in the low-CXI group had a significantly poorer OS than those in the high-CXI group (5-year OS: 63.4% vs. 78.5%, *P* < 0.001, Fig. 1a). Subdividing our cohort by pStage, patients with a low CXI consistently exhibited a poorer OS than those with a high CXI across all pStages (5-year OS: pStage I, 74.5% vs. 83.2%, *P* < 0.001; pStage II, 65.2% vs. 77.6%, *P* = 0.020; pStage III, 44.4% vs. 57.4%, *P* = 0.024; Fig. 1b-d).

**Fig. 2:**
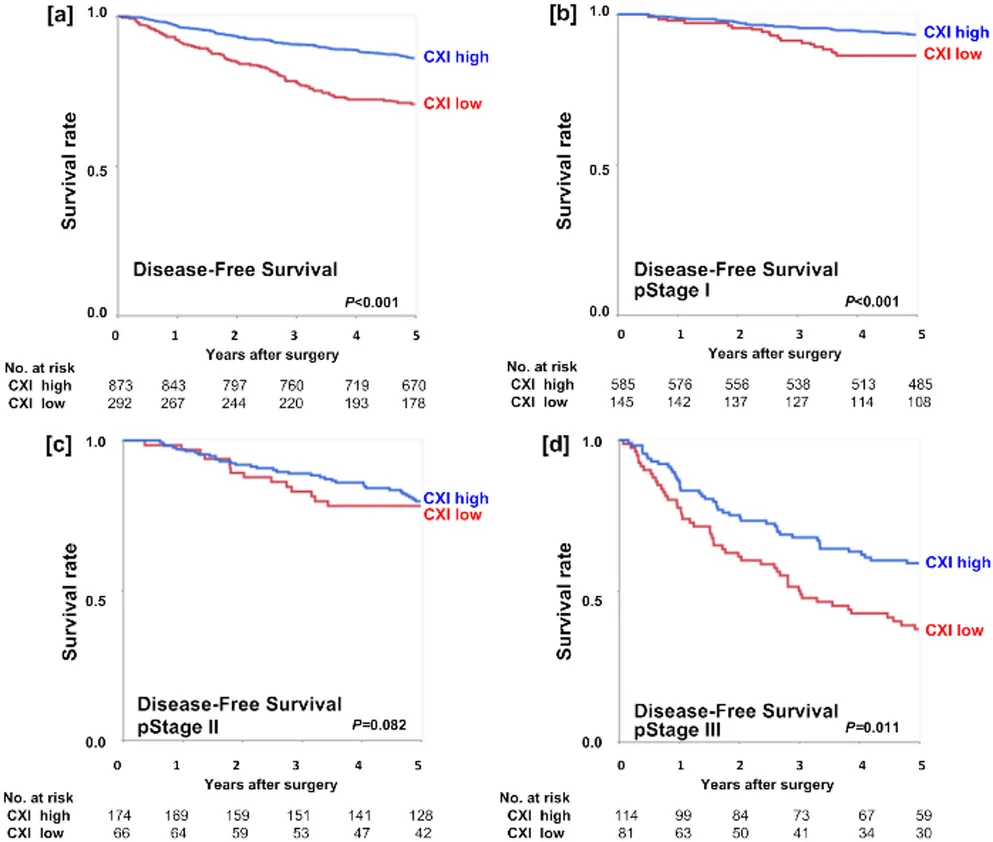
DFS outcomes according to the CXI A low CXI was significantly associated with a poor DFS **(a)** in the entire cohort (*P* < 0.001), **(b)** in patients with pStage I GC (*P* < 0.001), and **(d)** in patients with pStage III GC (*P* = 0.011)

Furthermore, the 5-year DFS of patients with a low CXI was significantly poorer than that of patients with a high CXI (61.0% vs. 76.7%, *P* < 0.001; Fig. 2a). The differences in DFS between the low- and high-CXI groups were also marked in patients with pStage I (74.5% vs. 82.9%, *P* < 0.001; Fig. 2b) and pStage III disease (37.0% vs. 51.8%, *P* = 0.011; Fig. 2d) as well as in those with pStage II-III disease (48.3% vs. 64.6%, *P* < 0.001; Supplementary Fig. 1). Taken together, a low CXI had a negative impact on the survival of GC patients, irrespective of pStage.

## Differences in recurrence patterns according to the CXI status

Given that patients with pStage I gastric cancer are at a very low risk of recurrence, we investigated recurrence patterns limited to patients with pStage II–III disease [28].

**Fig. 3:**
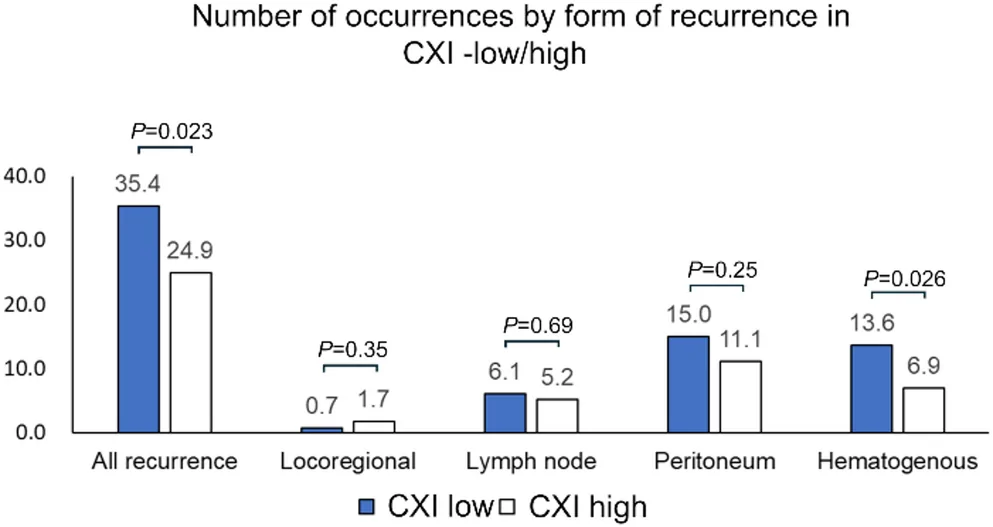
Recurrence patterns according to the CXI in patients with pStage II-III GC Among patients with pStage II-III, a low CXI was associated with a significantly higher incidence of recurrence (*P* = 0.023), particularly hematogenous recurrence (*P* = 0.026)

Among patients with pStage II–III GC, those in the low-CXI group had a significantly higher incidence of recurrence than those in the high-CXI group (35.4% vs. 24.9%, *P* = 0.023; Fig. 3). The locoregional, lymph node, and peritoneal recurrence rates were comparable between the low- and high-CXI groups (0.7% vs. 1.7%, *P* = 0.35; 6.1% vs. 5.2%, *P* = 0.69; 15.0% vs. 11.1%, *P* = 0.25; Fig. 3). Notably, the incidence of hematogenous recurrence was significantly higher in the low-CXI group than in the high-CXI group (13.6% vs. 6.9%, *P* = 0.026; Fig. 3). Overall, a low CXI was significantly associated with a higher incidence of recurrence, particularly hematogenous recurrence. (Details on the hematogenous recurrence pattern are presented in Supplementary Table 1)

**Table 1:** Characteristics of 1166 patients according to CXI

Variables	Low CXI	High CXI	P value
	(n=292)	(n=874)	
Age (years)			<0.001
Median (range)	70 (63–78)	65 (57–72)	
Sex			0.96
Male	204 (69.9)	612 (70.0)	
Female	88 (30.1)	262 (30.0)	
Comorbidity according to CCI			<0.001
CCI 0–1	231 (79.1)	773 (88.4)	
CCI ≧2	61 (20.9)	101 (11.6)	
Type of gastrectomy			<0.001
Total (including completion)	95 (32.5)	202 (23.1)	
Distal	138 (47.3)	404 (46.2)	
Proximal	10 (3.4)	28 (3.2)	
Pylorus-preserving	42 (14.4)	213 (24.4)	
Other	7 (2.4)	27 (3.1)	
Duration of hospital stay(days)			0.42
Median (range)	15(11–21)	14(11–19)	
Complications (≥ Grade II ^†^)	75 (25.7)	179 (20.5)	0.04
Tumor histology (Lauren)			0.06
Intestinal	99 (33.9)	328 (37.5)	
Diffuse	88 (30.1)	299 (34.2)	
Others	105 (36.0)	247 (28.3)	
pStage			<0.001
Stage I	145 (49.7)	585 (66.9)	
Stage II	66 (22.6)	174 (19.9)	
Stage III	81 (27.7)	115 (13.2)	

^†^ Clavien-Dindo classificationAbbreviations: CXI, Cachexia index; CCI, Charlson Comorbidity Index

### The CXI was an independent prognostic factor of a poor OS and DFS

Application of the Cox proportional hazards model revealed a low CXI (HR 1.58, 95% CI: 1.20–2.07, *P* < 0.001), age ≥ 65 years (HR 2.04, 95% CI: 1.51–2.75, *P* < 0.001), BMI < 18.5 (HR 1.61, 95% CI: 1.14–2.27, *P* = 0.007), CCI ≥ 2 (HR 1.73, 95% CI: 1.28–2.36, *P* = 0.004), total gastrectomy (HR 1.95, 95% CI: 1.49–2.54, *P* < 0.001), C-D classification ≥ 2 (HR 1.47, 95% CI: 1.12–1.93, *P* = 0.006), and pathological stage (I vs. II, *P* = 0.002; I vs. III, *P* < 0.001), to be independently associated with poor OS outcomes (Table 2).

**Table 2:** Cox hazards model for overall survival

Variables	Multivariable analysis		
	HR	95% CI	P
			value
Age≧65	2.04	1.51–2.75	<0.001
BMI<18.5	1.61	1.14–2.27	0.007
CCI≧2 (vs. 0–1)	1.73	1.28–2.36	0.004
Total gastrectomy	1.95	1.49–2.54	<0.001
Complication (≧GradeⅡ^†^)	1.47	1.12–1.93	0.006
pStageⅠ	Ref		
pStageⅡ	1.83	1.26–2.66	0.002
pStageⅢ	4.59	3.16–6.66	<0.001
Adjuvant (vs. non-Adjuvant)	0.75	0.54–1.04	0.09
low CXI (vs. high CXI)	1.58	1.20–2.07	0.001

^†^Clavien-Dindo classificationAbbreviations: CCI, Charlson Comorbidity Index; CXI, Cachexia Index

Notably, a multivariate Cox hazards model analysis focusing on DFS showed that a low CXI was an independent predictor of a poor DFS (HR 1.60, 95% CI: 1.24–2.07, *P* < 0.001) (Table 3).

**Table 3:** Cox hazards model for disease free survival

Variables	Univariable analysis			Multivariable analysis		
	HR	95% CI	P	HR	95% CI	P
			value			value
Age≧65	2.48	1.90–3.26	<0.001	1.94	1.47–2.56	<0.001
CCI≧2 (vs. 0–1)	1.96	1.46–2.61	<0.001	1.62	1.20–2.18	0.0014
Total gastrectomy	2.63	2.06–3.34	<0.001	1.77	1.38–2.29	<0.001
Complication (≧GradeⅡ^†^)	1.87	1.44–2.40	<0.001	1.37	1.06–1.78	0.018
Diffuse type (vs. non-Diffuse)	0.96	0.75–1.23	0.74			
pStageⅠ	Ref			Ref		
pStageⅡ	2.25	1.64–3.08	<0.001	1.96	1.42–2.70	<0.001
pStageⅢ	6.36	4.82–8.39	<0.001	4.8	3.59–6.42	<0.001
low CXI (vs. high CXI)	2.55	2.00–3.26	<0.001	1.6	1.24–2.07	<0.001

^†^Clavien-Dindo classificationAbbreviations: CCI, Charlson Comorbidity Index; CXI, Cachexia Index

Using the sex-specific median as the cutoff yielded less distinct separation between the CXI Low and High groups than quartile-based cutoffs. However, significant differences in the OS and DFS persisted (Supplementary Fig. 2, 3). Moreover, the CXI remained an independent factor when analyzed as a continuous variable in the multivariable analysis (Supplementary Table 2), thus supporting its robust prognostic value.

## Discussion

This study demonstrated that a reduced CXI was significantly associated with unfavorable short- and long-term outcomes in patients who underwent curative gastrectomy for primary GC. Notably, the prognostic impact of the CXI was consistently observed across all pathological stages, underscoring the robustness of the CXI as a survival indicator. Furthermore, patients with a low CXI exhibited a significantly higher incidence of hematogenous recurrence. Importantly, the CXI remained an independent predictor of overall survival, even after adjusting for established prognostic variables, including pathological stage, comorbidity burden, and surgical factors.

A low CXI was associated with advanced age, a higher CCI, a higher prevalence of postoperative complications, and an advanced pStage, findings consistent with prior investigations [17–19]. Malnutrition and progressive depletion of skeletal muscle mass are often observed in older populations [29, 30]. Cancer-associated cachexia has been suggested to impair systemic immune competence, potentially leading to an increased burden of comorbid conditions [31]. The prevalence of cachexia reportedly increases as the tumor stage advances, thereby accelerating disease progression [32]. Overall, the CXI potentially reflects the frailty of patients and tumor aggressiveness.

The present study identified total gastrectomy as an independent prognostic factor for the OS and DFS. Although this association may partly reflect surgical stress and cachexia in advanced disease, laparoscopic subtotal gastrectomy has been shown to yield better short-term outcomes and nutritional status than total gastrectomy [33]. These findings suggest that the poorer prognosis after total gastrectomy may reflect not only the operative burden and disease severity, but also an impaired postoperative nutritional status.

Patients with a low CXI developed postoperative complications more frequently than those with a high CXI. A recent study revealed that a low CXI is significantly associated with poor short-term outcomes after gastrectomy [34], although other studies showed no significant association between the CXI and postoperative complications in patients with gastrointestinal cancers [17, 18]. Given that sarcopenia and malnutrition are reportedly associated with a decline in cardiopulmonary reserve and immune competence [35, 36], the observed association between a low CXI and increased postoperative morbidity is plausible.

We also showed that a low CXI was significantly associated with a poor OS. The present investigation encompassed a relatively large patient population, whereas studies focusing specifically on gastric cancer within Asian cohorts remain limited [17]. Importantly, even after adjusting for established prognostic factors such as age, comorbidity, surgical procedure, postoperative complications, and pStage, a low CXI remained an independent predictor of a poor OS. These findings underscore the clinical relevance of preoperative cachexia-related physiological impairment in shaping the postoperative prognosis [18, 19].

Stage-stratified subgroup analyses revealed that a low CXI was associated with a poor OS in patients with both early and advanced GC, which is consistent with a recent study [37]. In early stage GC, non-cancer-related mortality often determines survival after gastrectomy [3–6]. Our findings suggest that the higher prevalence of malnutrition and sarcopenia among patients with a low CXI may partially explain the increased risk of non-cancer-related mortality. The CXI potentially represents frailty in GC patients, thereby influencing non-GC-related deaths in the tumor population [38–41]. The survival impact of low CXI was observed even in advanced GC. A recent study suggested that the combination of sarcopenia, systemic inflammation, and malnutrition sharply reflects tumor biological aggressiveness [11, 42]. Overall, the CXI potentially reflects the combined influence of the tumor burden and frailty-related physiological deterioration.

A recent study suggested that a low CXI is associated with recurrence patterns in patients undergoing neoadjuvant chemotherapy and surgery for GC [43]. However, the association between a low CXI and recurrence patterns remains to be investigated in patients who underwent upfront surgery for GC. We revealed that patients with a low CXI were at a higher risk of hematogenous recurrence than those with a high CXI. Systemic immunosuppression reportedly attenuates antitumor immune responses, leading to poor oncological outcomes [44]. Specifically, it has been reported that patients with a low Prognostic Nutritional Index (PNI) tend to exhibit reduced levels of CD8-positive lymphocytes [45]. In addition, Doi et al. reported that among patients with hepatocellular carcinoma (HCC), those with sarcopenia had a significantly poorer recurrence-free survival (RFS), accompanied by a decreased infiltration of CD4-positive, CD8-positive, and CD45RO-positive lymphocytes [46]. These findings suggest that both impaired local immune responses and compromised systemic immunity may contribute to unfavorable oncologic outcomes. The correlation between systemic immunity and local tumor immunity may underlie the higher incidence of hematogenous recurrence in patients with a low CXI. However, as a direct causal relationship was not demonstrated in this study, further investigation into the underlying mechanisms is warranted. This study showed that GC patients with a low CXI experienced a substantially elevated incidence of postoperative recurrence, suggesting a possible association between CXI and the selection of postoperative adjuvant chemotherapy.

Preoperative CXI assessment may assist clinicians in identifying patients who may benefit from targeted prehabilitation strategies. Preoperative rehabilitation and nutritional support may be associated with improvements in muscle strength and nutritional status, which may, in turn, be related to favorable survival outcomes in patients with a low CXI [18]. However, as these interventions were not evaluated in this study, their clinical benefits require confirmation in prospective studies. Although preoperative exercise and nutritional interventions are reportedly useful for improving the short-term outcomes [47, 48], it remains controversial whether these interventions improve the long-term survival outcomes of patients with cancer [49, 50]. The CXI can be calculated using routinely obtained laboratory and imaging data before surgery; however, further prospective studies are required to assess the clinical utility of the CXI.

This study has some limitations. First, this was a single-institution, retrospective study. Our results must be carefully interpreted in the context of the high rate of pStage I disease in our cohort. Specifically, the large proportion of patients with early stage disease, in whom the influence of tumor-related cachexia is likely limited, may have reduced the ability of the CXI to discriminate clinical outcomes. Therefore, additional studies must be conducted to validate the utility of this score in an external independent cohort. Second, the optimal thresholds for the CXI have not yet been established. In this study, a low CXI was defined as a value within the lowest quartile, stratified by sex. Prior investigations have assessed the prognostic significance of the CXI using various methods, including median-based thresholds and a receiver operating characteristic curve analysis [17, 36]. To support the wider clinical implementation of the CXI, additional studies are required to establish robust analytical approaches, including the determination of appropriate cutoff values.

In conclusion, the CXI may serve as a potentially useful prognostic indicator in patients undergoing surgery for GC, as it is associated with both the OS and DFS. The CXI may reflect both patient-related physiological factors and tumor-related characteristics and it was associated with postoperative survival across different cancer stages in this study.

## Electronic Supplementary Material

Below is the link to the electronic supplementary material.

Supplementary material 1

Supplementary material 2

Supplementary material 3

## Abbreviations

CXI: Cachexia Index
GC: Gastric cancer
SMI: skeletal muscle index
NLR: neutrophil-to-lymphocyte ratio
OS: overall survival
DFS: disease-free survival
PNI: prognostic nutritional index
CCI: Charlson Comorbidity Index
C-D: Clavien-Dindo
