# Supplementary material for: Impact of the Cachexia Index on survival and recurrence in patients undergoing surgery for gastric cancer
Source: Surg Today. 2026 Jul 13;56(11):2667–74. doi: 10.1007/s00595-026-03393-y (PMC13722633; doi:10.1007/s00595-026-03393-y)
Supplement: Supplementary file 1 [file 595_2026_3393_MOESM3_ESM.docx]

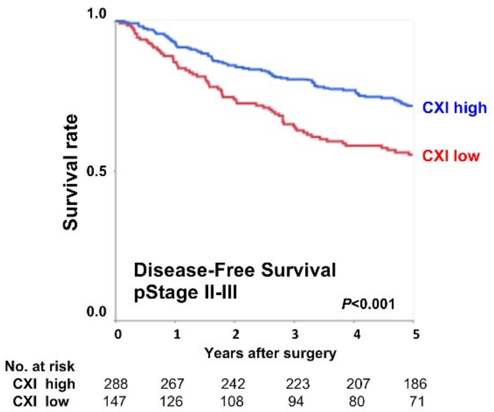

Supplementary Figure1. DFS outcomes according to CXI

Low CXI was significantly associated with poor DFS in the patients with pStage II-III GC (P<0.001).

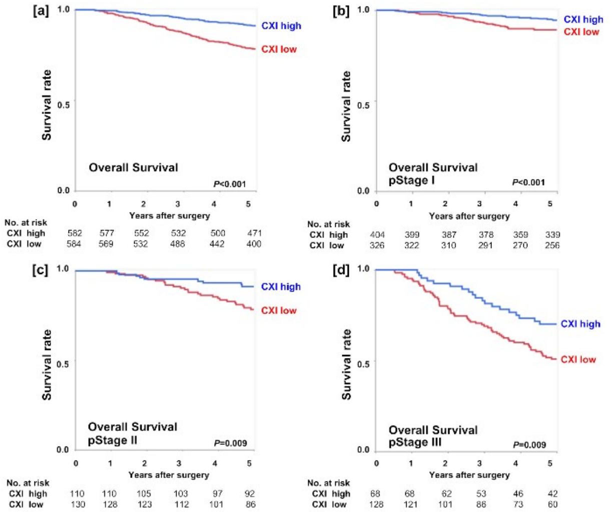

Supplementary Figure2. OS outcomes according to CXI (by sex-specific median)

(a) Patients in the low-CXI group exhibited significantly poorer OS than those in the high-CXI group (P<0.001). The survival difference according to CXI was also found in patients with (b) pStage I GC (P < 0.001), (c) pStage II GC (P = 0.009), and (d) pStage III GC (P = 0.009).

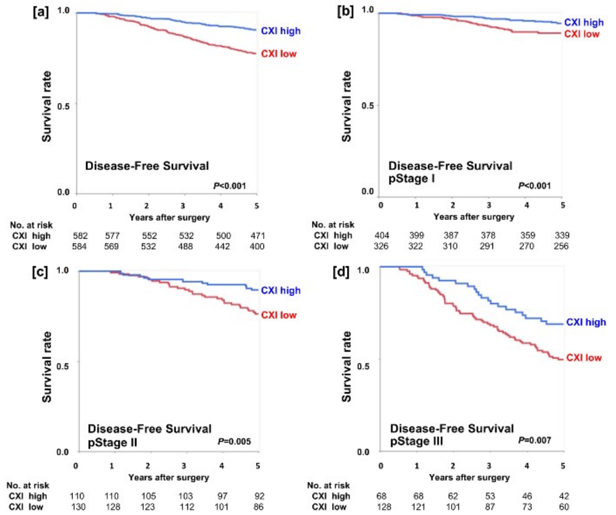

Supplementary Figure3. DFS outcomes according to CXI (by sex-specific median)

(a) Patients in the low-CXI group exhibited significantly poorer DFS than those in the high-CXI group (P<0.001). The survival difference according to CXI was also found in patients with (b) pStage I GC (P < 0.001), (c) pStage II GC (P = 0.005), and (d) pStage III GC (P = 0.007).
