# Supplementary material for: Impact of the Cachexia Index on survival and recurrence in patients undergoing surgery for gastric cancer
Source: Surg Today. 2026 Jul 13;56(11):2667–74. doi: 10.1007/s00595-026-03393-y (PMC13722633; doi:10.1007/s00595-026-03393-y)
Supplement: Supplementary file 1 [file 595_2026_3393_MOESM1_ESM.docx]

| **Supplementary Table1.**  **Details on the Hematogenous Recurrence Pattern** | |
| --- | --- |
| Site of recurrence | Number of patients (%) |
| Liver  Bone  Abdominal wall  Lung  Brain  Spleen  Multiple metastases^†^ | 23(48.9)  5(10.6)  4(8.5)  2(4.2)  1(2.1)  1(2.1)  11(23.4) |
| ^†^Multiple metastases: Metastases were detected in two or more organs. | |
