# Supplementary material for: Impact of the Cachexia Index on survival and recurrence in patients undergoing surgery for gastric cancer
Source: Surg Today. 2026 Jul 13;56(11):2667–74. doi: 10.1007/s00595-026-03393-y (PMC13722633; doi:10.1007/s00595-026-03393-y)
Supplement: Supplementary file 2 [file 595_2026_3393_MOESM2_ESM.docx]

| **Supplementary Table 2. Cox hazards model for overall survival** | | | |
| --- | --- | --- | --- |
| Variables | Multivariable analysis | | |
|  | HR | 95% CI | *P*  value |
| Age≧65  CCI≧2 (vs. 0-1)  Total gastrectomy  Complication (≧GradeⅡ^†^)  Diffuse type (vs. non-Diffuse)  pStageⅠ  pStageⅡ  pStageⅢ  CXI (Continuous variables) | 2.08  1.82  1.92  1.52  Ref  1.62  3.91  0.99 | 1.55-2.80  1.35-2.47  1.48-2.51  1.16-1.99  1.15-2.27  2.90-5.28  0.99-1.00 | <0.001  <0.001  <0.001  0.004  0.006  <0.001  <0.001 |
| ^†^ Clavien-Dindo classification  Abbreviations: CCI, Charlson Comorbidity Index; CXI, Cachexia Index | | | |
